# Listeriosis during pregnancy: a retrospective cohort study

**DOI:** 10.1186/s12884-022-04613-2

**Published:** 2022-03-28

**Authors:** Yefang Ke, Lina Ye, Pan Zhu, Ying Sun, Zhe Zhu

**Affiliations:** 1 Department of Clinical Laboratory, Ningbo Women and Children’s Hospital, Ningbo, Zhejiang China; 2 Neonatal Intensive Care Unit, Ningbo Women and Children’s Hospital, Ningbo, Zhejiang China; 3 Department of Obstetrics, Ningbo Women and Children’s Hospital, Ningbo, Zhejiang China; 4 Department of Blood Transfusion, HwaMei Hospital, University of Chinese Academy of Sciences, Ningbo, Zhejiang China; 5 Ningbo Institute of Life and Health Industry, University of Chinese Academy of Sciences, Ningbo, Zhejiang China

**Keywords:** Pregnancy-associated listeriosis, *Listeria monocytogenes*, Maternal listeriosis, Neonatal listeriosis, Outcome

## Abstract

**Background:**

Pregnancy-associated listeriosis is a severe infectious disease and potentially leads to fetal/neonatal fatal, while limited information on pregnancy-associated listeriosis is available in China. This study aimed to reveal the clinical characteristics and outcomes of pregnancy-associated listeriosis cases and provide references for treating and managing this disease.

**Methods:**

We performed a retrospective study on maternal and neonatal patients with pregnancy-associated listeriosis. The clinical characteristics of pregnancy-associated listeriosis were studied, and the outcome determinants of neonatal listeriosis were explored.

**Results:**

14 cases of pregnancy-associated listeriosis were identified. The incidence of pregnancy-associated listeriosis in our hospital was 16.69/100,000 births. All of the 14 maternal patients eventually recovered after delivery shortly with no sequelae. None of the 12 mothers who delivered in this hospital received antepartum first-line empirical treatment. Among the 14 neonatal cases, 1 was late-onset listeriosis and 13 were early-onset cases; 11 survived and 3 died. Fatality rates were significantly higher in outborn neonates (*P* = 0.005). Besides, higher mortality rates were observed in neonates with lower birth weight (*P* = 0.038), gestational age < 28 weeks (*P* = 0.056), and Apgar score (5^th^ min) < 5 (*P* = 0.056), with marginally significant differences.

**Conclusions:**

Pregnancy-associated listeriosis would bring disastrous effects to the neonatal cases, especially to the outborn, low birth weight, and low gestational age of neonates. Timely detection and treatment should be taken seriously for the key neonates. How to early detect *L. monocytogenes* infected cases, especially in the prenatal stage, remains a serious challenge.

## Background

Listeriosis is a rare but severe disease caused by *Listeria monocytogenes* (*L. monocytogenes*), a ubiquitous food-borne bacterial pathogen [1, 2]. People principally get listeriosis by the ingestion of contaminated food [3, 4]. Especially, it has a predilection to infect pregnant women, which is at approximately 10–100 times greater risk for infection than the general population [5–7]. In Europe and North America, it has been observed previously that the proportion of pregnancy-associated listeriosis out of the total listeria infections ranges from 9 to 20% [2, 8–10]. Moreover, recent studies performed in China showed that more than half of listeria cases were pregnancy-associated infections [11, 12].

The high incidence of listeriosis during pregnancy may be due to the increased progesterone levels which would affect immune functions [13, 14]. *L. monocytogenes* can actively cross the intestinal barrier, disseminate within the circulation, cross the placental barrier owing to its specific placental tropism, and eventually lead to placental and fetal infections [15]. Although the maternal illness is usually mild, neonatal illness is frequently severe and potentially fatal. Pregnancy-associated listeriosis can lead to miscarriage, preterm delivery, or stillbirth. It was reported that more than 80% of infected mothers would experience major fetal or neonatal complications [9]. Therefore, pregnancy-associated listeriosis is recognized as one of the worst maternal infections.

It has been reported that the incidence of pregnancy-associated listeriosis varied between different minorities [4, 9, 16]. Recently, a study in Singapore found that the incidence of congenital listeriosis was highest in infants of Chinese origin [17]. Besides, a study observed that the rate of pregnancy-associated listeriosis in Beijing Obstetrics and Gynecology Hospital was 13.7 per 100,000 births [18], which was higher than that in the United States, United Kingdom, and New Zealand (3.4, 3.4 and 12.3 per 100,000 births, respectively) [19–21]. Listeriosis has not yet been included in the notifiable disease in China, the surveillance of listeriosis has not been thoroughly implemented. Moreover, owing to the nonspecific obstetrical signs and long incubation period, the diagnosis of pregnancy-associated listeriosis would be challenging [22, 23].

To better understand the maternal and neonatal listeriosis in Ningbo, Zhejiang Province, China and to provide more information for formulating appropriate therapeutic and controlling strategies, we performed a retrospective study to analyze the clinical characteristics and outcomes of pregnancy-associated listeriosis cases.

## Methods

### Study design and patient selection

This was a retrospective study conducted at Ningbo women and children’s hospital, a tertiary maternity and pediatric hospital, where the number of births is about 10,000 every year. This study was approved by the institutional ethics board (EC2021-028), and the informed consent was waived for this retrospective study. All pregnancy-associated listeriosis cases from August 2013 to September 2021 were included in this retrospective study.

Blood cultures were performed in the pregnant women with the severe performance of infections, and amniotic fluid and placental tissue cultures were performed in the women with suspected intrauterine infections; while blood cultures were routinely conducted in every newborn on admission and were conducted again when they had symptoms of infection during hospitalization, gastric fluid cultures were conducted in the newborns with suspected intrauterine infections, and bacterial cultures of other sites where were suspected to be infected would be done at the discretion of the physicians. Positive for isolation of *L. monocytogenes* from a normally sterile site of the pregnant women and/or newborns aged ≤ 28 days, were defined as confirmed cases. In our hospital, management of women with symptoms of infections during the perinatal period was in preference to receive cephalosporins as an empirical therapy which depended on the discretion of the physicians, whether listeriosis was later found or not; the empirical antibiotics therapy in children was determined by the physicians based on clinical symptoms and the characteristics of the infections in their mothers.

Data on past medical history, characteristics at admission, underlying medical conditions, laboratory results, and treatments were collected from the medical record database.

### Definitions

A pregnancy-associated listeriosis case was defined based on the isolation of *L. monocytogenes* from a normally sterile site of the pregnant women and/or newborns aged ≤ 28 days. Each Mother and her newborn were counted as a single case. Neonatal listeriosis cases were classified as early-onset (diagnosed between birth and days 6) listeriosis or late-onset (diagnosed between days 7 and 28) listeriosis. We defined “inevitable miscarriage” as fetal loss before 24 weeks of gestation, “stillbirth” as the death of the fetus between 24 and 41 weeks of gestation, “maternal leukocytosis” as a white blood cell (WBC) count > 12 × 10^9^/L, “neonatal leukocytosis” as a WBC count > 34 × 10^9^/L, “neonatal leukopenia” as a WBC count < 5 × 10^9^/L, “neonatal thrombocytopenia” as a platelet count < 150 × 10^9^/L, “neonatal severe thrombocytopenia” as a platelet count < 5 × 10^9^/L, “inborn neonate” as a neonate born in our hospital, and “outborn neonate” as a neonate born in other hospitals and transferred to our hospital after birth.

### Microbiological methods

For blood culturing, whole blood for bacterial culture was collected and incubated in an automatic blood culture system (BACT/ALERT 3D) for 5 days or until rated positively. Positive blood culture was incubated on blood agar at 36℃ for 18–24 h. CSF, cervical secretion, eye secretion, gastric aspirate of newborn, and products of conception samples were cultured directly on blood agar at 36℃ for 18–48 h, then, bacteria with a positive growth were isolated and cultured onto blood agar at 36℃ for 18–24 h.

The identification of bacteria was performed by using the VITEK 2 COMPACT automatic analysis system (BioMérieux, France). Standard strains (ATCC700323, ATCC25922, ATCC700327, ATCC29213) were used to control the microbiological quality.

### Statistical analysis

Clinical, laboratory, and meteorology data were recorded in a Microsoft Excel database (Microsoft, Richmond, US). For continuous variables, non-normally distributed variables were expressed as medians and ranges; for categorical variables, data were presented as percentages. The differences between the two groups were determined by using the Wilcoxon Manne-Whitney U test for quantitative data and Fisher’s exact test for qualitative data, respectively. A *P* < 0.05 was considered statistically significant. All statistical analyses were performed by using SPSS statistical 22.0 software (IBM, Armonk, NY, USA).

## Results

### Epidemiological characteristics of pregnancy-associated listeriosis cases

During the study period, 14 cases of pregnancy-associated listeriosis were identified. 12 cases were inborn neonates, the rest (B2 and B3) were outborn neonates. The incidence of pregnancy-associated listeriosis in our hospital was 16.69/100,000 (14/83,875) births during our study period. They occurred in all seasons, and half were occurred in summer (50.0%, 7/14).

### Characteristics of maternal cases

The characteristics of the 14 maternal listeriosis cases of pregnancy-associated listeriosis were summarized in Table 1. Among them, 13 (M1-11, 13, 14) were singleton pregnancies and 1 (M12) was a twin pregnancy. Positive *L. monocytogenes* cultures from blood, amniotic fluid, or placental tissue samples were found in eight (57.1%, 8/14) mothers.

**Table 1:** Characteristics of maternal listeriosis cases

No	Gestation	Clinical presentation	Culture sites			MSAF	WBC (10^9^/L)	CRP (mg/L)	Mode of delivery	Antibiotic(s) Before Delivery	Admission-delivery interval(hour/s)	Concomitant disease	Maternal outcome	Neonatal/fetal outcome
			Blood	Amniotic fluid	Placental tissue									
M1	38	None	/	/	/	No	8.5	4.7	Cesarean section	Cefuroxime	24	Pelvic adhesions	Recovered	Survived
M2	32	NA	NA	NA	NA	NA	NA	NA	Vaginal delivery	NA	NA	NA	NA	Death, neonatal listeriosis
M3	26	NA	NA	NA	NA	NA	NA	NA	Vaginal delivery	NA	NA	NA	NA	Death, neonatal listeriosis
M4	30	Fever Tmax 38.2℃, Abd pain, vaginal bleeding	/	/	/	Yes	19.0	25	Vaginal delivery	Cefuroxime	22.5	GDM, HSP	Recovered	Survived, neonatal listeriosis
M5	29	Abd pain, vaginal bleeding, RFM	/	/	/	Yes	13.9	54.4	Vaginal delivery	No	4	Chorioamnionitis	Recovered	Survived, neonatal listeriosis
M6	34	RFM	/	/	/	Yes	17.3	30	Cesarean section	Azithromycin	3.5	Thrombophilia, chorioamnionitis	Recovered	Survived, neonatal listeriosis
M7	30	Abd pain, vaginal bleeding	/	( +)	( +)	Yes	27.6	56.8	Cesarean section	Piperacillin and sulbactam	10	URI, HBV carrier, Ovarian teratoma	Recovered	Survived, neonatal listeriosis
M8	30	Fever Tmax 39.7℃	( +)	( +)	( +)	Yes	17.0	52.9	Cesarean section	Ceftriaxone	1	GBS colonization, chorioamnionitis, sepsis, moderate anemia, Gallbladder polyps	Recovered	Survived, neonatal listeriosis
M9	37	Abd pain	( +)	/	/	No	7.1	64	Cesarean section	Cefuroxime	1	Chorioamnionitis, sepsis	Recovered	Survived, neonatal listeriosis
M10	28	Abd pain, ROM, flu-like symptoms	/	( +)	( +)	Yes	24.8	62.8	Cesarean section	Ceftriaxone	1.5	Chorioamnionitis,	Recovered	Death, neonatal listeriosis
M11	32	Abd pain, ROM	/	( +)	/	No	23.6	35	Cesarean section	Piperacillin and sulbactam + Metronidazole	5.5	No	Recovered	Survived
M12	36	Fever Tmax 38.4℃	/	(-)	( +)	No	24.0	85.2	Cesarean section	Cefotaxime	4	Chorioamnionitis, hyperthyroidism, polycystic ovarian syndrome	Recovered	Survived
M13	18	Fever Tmax 38℃, Abd pain, ROM, vaginal bleeding	(-)	( +)	/	No	17.2	96.9	Inevitable miscarriage	Piperacillin and sulbactam	17.5	Chorioamnionitis, Mild anemia	Recovered	Intrauterine fetal death
M14	35	Fever Tmax 38℃, Abd pain	(-)	( +)	( +)	Yes	23.1	36.3	Cesarean section	Ceftriaxone	3.5	Bilateral mesosalpinx cyst	Recovered	Survived

*MSAF* meconium-stained amniotic fluid, *WBC* white blood cell, *CRP* C-reactive protein, *NA* not available, *Tmax* maximal temperature, *Abd* pain abdominal pain, *GDM* gestational diabetes mellitus, *HSP* hereditary spastic paraplegia, *RFM* reduced fetal movements, *URI* upper respiratory infection, *HBV* hepatitis B virus, *GBS* Group B *streptococcus*, *ROM* rupture of membranes

Two cases were infected with *L. monocytogenes* in the second-trimester pregnancy (between 14 and 27 weeks), and others were infected in the trimester pregnancy (between 28 and 41 weeks). Their median gestational age at the time of infection was 31 weeks (range: 18–38 weeks). Among the 14 maternal patients, 12 had premature deliveries, 1 experienced intrauterine fetal death, and 1 had term delivery.

One of the 14 maternal patients experienced inevitable miscarriage, 4 had nature labor; and cesarean sections were performed in 9 cases, 8 due to fetal distress or intrauterine infection, 1 due to scarred uterus. Among the 12 mothers who delivered in this hospital, 41.7% (5/12) had prenatal fever at admission, only one patient (1/12, 8.3%) had flu-like symptoms before delivery. No patient with gastrointestinal symptoms before delivery has been observed, and only one patient had diarrhea after delivery. Various obstetrical symptoms were observed, including abdominal pain (8/12, 66.7%), a small amount of vaginal bleeding (4/12, 33.3%), which was due to abruption, rupture of membranes (9/12, 75.0%), and reduced fetal movement (2/12, 16.7%).

83.3% (10/12) maternal listeriosis patients had leukocytosis, and 91.7% (11/12) had elevated CRP levels. 58.3% (7/12) maternal cases had meconium-stained amniotic fluid (MSAF), and 58.3% (7/12) had chorioamnionitis. 91.7% (11/12) mothers were treated with pre-delivery antibiotics, but none received antepartum first-line empirical treatment (ampicillin or in combination with gentamicin). All of them eventually recovered after delivery shortly with no sequelae. However, the perinatal data of two maternal patients who delivered outside this hospital were unacquirable for analysis.

### Characteristics of the neonatal cases

The characteristics of 14 neonatal listeriosis cases were summarized in Table 2. There were 11 culture-confirmed cases and 3 probable cases, most of them were premature infants (85.7%, 12/14). The majority of neonatal cases were treated with Penicillin or Cephalosporin or Meropenem empirically.

**Table 2:** Characteristics of neonatal cases

No	Sex	GA (wk)	BW (g)	Apgar^a^	Clinical presentation	Culture sites			diagnosed day (postnatal)	Intubation	WBC (10^9^/L)	PLT (10^9^/L)	Empirical antibiotic treatment	Complications	Outcome
						Blood	Gastric fluid	Others							
B1	F	38	3760	9–10	Fever Tmax 38.1℃	( +)	/	/	12	No	8	171	Amoxicillin and Clavulanate Potassium	Septicemia, patent foramen ovale	A
B2	F	32	1650	5–8	RD, apnea, cyanosis, petechiae	( +)	/	Catheter ( +)	5	No	9.6	220	Azlocillin	Abnormal liver function, pneumonia, septicemia, preterm birth, low birth weight	D
B3	F	26	1000	4–4	RD, apnea, petechiae, rash, cyanosis	( +)	/	/	4	Yes	6.3	103	Penicillin	Pneumonia, extremely premature infant, very low birth weight	D
B4	M	30	1650	5–7	RD, apnea, cyanosis	( +)	( +)	Eye secretions ( +)	5	Yes	3.0	100	Penicillin + Meropenem	Septicemia, purulent meningitis, pneumonia, congenital heart disease, neonatal anemia, BIPI, neonatal intracranial hemorrhage, premature infant, low birth weight	A
B5	F	29	1350	7–8	RD, apnea, hypoglycemia, cyanosis, DOIC 5 min	( +)	( +)	Sputum ( +)	4	Yes	18.4	111	Cefotiam + Penicillin	Pneumonia, septicemia, purulent meningitis, neonatal anemia, BPD, hypoglycemia, premature infant, very low birth weight, apnea neonatorum	A
B6	M	34	2250	1–6	RD, apnea, lethargy, DOIC 5 min	( +)	( +)	/	4	Yes	9.2	177	Penicillin + Ceftazidime	Sepsis, Pneumonia, PDA, preterm birth, low birth weight	A
B7	F	30	1400	6–9	RD, apnea, DOIC 1 min	( +)	( +)	/	5	Yes	12.3	186	Penicillin + Meropenem	Pneumonia, septicemia, purulent meningitis, anemia, ROP, preterm birth, low birth weight	A
B8	M	30	1900	5–7	RD, apnea, cyanosis, DOIC 5 min	( +)	( +)	/	4	Yes	9.3	35	Vancomycin + Meropenem	Sepsis, shock, respiratory failure, cardiac insufficiency, AKI, capillary leak syndrome, purulent meningitis, intracranial hemorrhage, seizure, patent ductus arteriosus, coagulation disorders, thrombocytopenia, anemia, low birth weight, preterm birth, pneumorrhagia, hypoproteinemia, pneumonia, hypernatremia	A
B9	F	37	2700	9–10	Lethargy, rash	( +)	/	/	5	Yes	1.5	115	Penicillin + Ceftazidime	Sepsis, septic shock, respiratory failure, pneumonia, acidosis, coagulation disorders, thrombocytopenia, hypoproteinemia, seizure, hypocalcemia, purulent meningitis, acute renal injury	A
B10	M	28	1250	8–9	RD	(-)	( +)	/	5	Yes	13.5	140	Penicillin + Meropenem	Respiratory failure, pneumonia, septicemia, very low birth weight, preterm birth, patent ductus arteriosus, hypoproteinemia, anemia, congenital hypothyroidism	D
B11	F	32	1600	9–10	RD	(-)	/	/	5	No	19.6	350	Azlocillin	Pneumonia, preterm birth, low birth weight	A
B12.1	F	36	2550	9–10	RD, lethargy	(-)	/	/	5	No	19.6	236	Ceftazidime	Pneumonia, preterm birth	A
B12.2	M	36	2250	9–10	RD, lethargy, petechiae	(-)	/	/	5	No	16.2	223	Ceftazidime	Pneumonia, preterm birth, low birth weight	A
B13	M	35	2570	9–10	Cyanosis	(-)	( +)	/	6	No	17.5	230	Penicillin + Ceftazidime	Pneumonia, preterm birth, septicemia, neonatal hypocalcemia, neonatal anemia, neonatal hypokalemia, ASD	A

^a^Apgar score at 1^st^ min—5^th^ min

*GA* gestational age, *BW* birth weight, *PLT* platelet, *A* alive, *RD* respiratory distress, *D* dead, *BIPI* brain injure in premature infants, *DOIC* delay of initial crying, *BPD* bronchopulmonary dysplasia, *PDA* patent ductus arteriosus, *ROP* retinopathy of prematurity, *AKI* acute kidney injury, *ASD* atrial septal defect

### Culture-confirmed neonatal listeriosis cases

In the 11 culture-confirmed cases, blood and newborn gastric fluid were the sensitive samples for microbiological diagnosis (positive in 9 [81.8%] of 11 blood samples and 7 [100.0%] of 7 gastric fluid samples); 9 (81.8%) were inborn neonates and 2 (18.2%) were outborn neonates; 6 (54.5%) were female and 5 (45.5%) were male. Most of them were preterm infants (9/11, 81.8%), with a median gestational age (GA) of 30 weeks (range: 26–38 weeks) and a median birth weight (BW) of 1650 g (range: 1000-3760 g), 8 (72.7%) weighed < 2500 g and 4 (36.4%) weighed < 1500 g.

Most of the 11 confirmed neonatal listeriosis cases were early-onset (90.9%, 10/11) (B2-B10, B13). Among the 10 early-onset cases, 8 cases (80.0%, 8/10) had respiratory distress and 7 cases (70.0%, 7/10) received resuscitation at birth. The late-onset one was a female who was delivered by cesarean section at GA of 38 weeks and admitted to hospital with fever for one day at 8 days after birth.

Laboratory analyses were performed immediately on admission. Thrombocytopenia was observed in 54.5% (6/11) of culture-confirmed cases. Cerebrospinal fluid tap was performed in 81.8% (9/11) of neonates, and 4 of them were diagnosed with purulent meningitis, however, their CSF cultures were all negative. Cranial imaging revealed intracranial hemorrhage in 2 neonates (B4 and B8), and one of them (B4) was diagnosed as brain injury in premature infants (BIPI).

All of the neonates with confirmed listeriosis were treated empirically, and most of them were treated with Penicillin or Cephalosporin, or Meropenem. Eight newborns received intubation. Three neonates (B2, B3, and B10) died, and two of them were outborn.

### Probable neonatal listeriosis cases

Three newborns (B11, B12.1, and B12.2) were identified as probable neonatal listeriosis cases, including a pair of twins (B12.1 and B12.2). Both of their mothers were confirmed maternal listeriosis, but the culture results for these three neonates were negative. All of them were preterm infants, with GA of 32, 36, and 36 weeks, and BW of 1600 g, 2550 g, and 2250 g, respectively. They all had respiratory distress at birth and were diagnosed with pneumonia subsequently, their laboratory parameters were mildly abnormal. All of them were recovered soon after birth.

### Risk factors for mortality in neonates with pregnancy-associated listeriosis

We compared the characteristics of the surviving neonates (*n* = 11) with those of the non-surviving neonates (*n* = 3), trying to identify risk factors for mortality in neonates with pregnancy-associated listeriosis (Table 3).

**Table 3:** Comparison between the survival and fatal cases of pregnancy-associated listeriosis

	**Survival** **(*n* = 11)**	**Fatality** **(*n* = 3)**	***P* value**
BW			
BW (g)^a^	2250 (1600–2570)	1250 (1000–1650)	0.038
VLBW < 1500 g	2 (18.2%)	2 (66.7%)	0.112
GA			
GA (weeks)^a^	34 (30–36)	28 (26–32)	0.060
GA < 28 weeks	0 (0.0%)	1 (33.3%)	0.056
Apgar score			
Apgar score (1^st^min)^a^	9 (5–9)	5 (4–8)	0.225
Apgar score (1^st^ min) < 5	1 (9.1%)	1 (33.3%)	0.305
Apgar score (5^th^min)^a^	10 (7–10)	8 (4–9)	0.225
Apgar score (5^th^ min) < 5	0 (0.0%)	1 (33.3%)	0.056
Outborn (n, %)	0 (0.0%)	2 (66.7%)	0.005
Clinical Presentation			
Respiratory distress	8 (72.7%)	3 (100.0%)	0.325
Apnea	5 (45.5%)	2 (66.7%)	0.530
Cyanosis	3 (27.3%)	2 (66.7%)	0.224
Laboratory parameters			
WBC, 10^9^/L^a^	12.3 (8.0–18.4)	9.6 (6.3–13.5)	0.659
PLT, 10^9^/L^a^	177 (111–230)	140 (103–220)	0.659

^a^Median(ranges)

*BW* birth weight, *VLBW* very low birth weight, *GA* gestational age, *WBC* white blood cell, *PLT* platelet

Fatality rates were significantly higher in outborn neonates (*P* = 0.005). Besides, higher mortality rates were observed in neonates with lower BW (*P* = 0.038), GA < 28 weeks (*P* = 0.056), and Apgar score (5^th^ min) < 5 (*P* = 0.056), with marginally significant differences.

## Discussion

To our knowledge, this is the first detailed study of pregnancy-associated listeriosis infections among children in Ningbo, Zhejiang province, China. The incidence of pregnancy-associated listeriosis in our hospital was 16.69/100,000 deliveries, most of the neonatal listeriosis cases (92.9%, 13/14) were early onset. 3 cases died, all of them were early-onset and culture-confirmed neonates, 2 of them were outborn neonates. All of the maternal patients recovered after delivery shortly with no sequelae.

The diagnosis of maternal listeriosis would be challenging, apart from nonspecific obstetrical signs, fever or flu-like symptoms are the only clinical signs in infected mothers [24], but they were not always present. In our study, only one patient had flu-like symptoms before delivery. The difficulty of diagnosing was associated with adverse fetal or neonatal outcomes. First, we and others all observed that most maternal listeriosis cases were appeared during the second and third trimester period of pregnancy, mainly based on the adverse signs of their infants. While the occurrence of first-trimester maternal listeriosis was likely underestimated as the non-specific nature of disease presentation, thus may causing early fetal losses. Second, non-specific signs in infected pregnant women would influence antibiotic prophylaxis to prevent neonatal listeriosis [22]. Previous studies have shown that adequate maternal antimicrobial treatment before delivery was associated with a significant decrease in infants’ severity [24, 25]. Unfortunately, in our study and others performed in China [18], none of the maternal listeriosis cases received adequate first-line antimicrobial treatment [9]. This may partly account for the adverse outcome of *L. monocytogenes* infected neonates in the present study. In China, there are no national guidelines for the treatment of listeriosis currently. Although rare, listeriosis should be considered in the differential diagnosis of pregnancy-associated infections.

Pregnant women have much higher rates of *L. monocytogenes* infection than the general population [5–7]. Meanwhile, the consequences of listeriosis during pregnancy and in neonates are often severe [9]. Listeriosis neonates are always in critical condition, especially the early-onset ones [15]. In the present study, 80.0% (8/10) of early-onset confirmed neonatal listeriosis cases had respiratory distress, 70.0% (7/10) received resuscitation at birth, and 30.0% (3/10) died. Identified risk factors for mortality in neonates with pregnancy-associated listeriosis is important to treat and manage the high-risk neonates. It was found that fatality rates were significantly higher in the neonates who were born in other hospitals (*P* = 0.005) in our study, these hospitals were lack of neonatal intensive care units. It has been observed in a previous study that preterm neonates, who were delivered in hospitals with lower levels of neonatal care, suffered from higher rates of adverse outcomes [26]. The capability to provide timely and optimal resuscitative measures is important to reduce mortality of neonatal listeriosis.

Previous studies have also shown that lower GA was associated with higher mortality [9]. While, later infection, particularly which occurred in the third trimester, is typically associated with more favorable outcomes than earlier infection. In our study, higher mortality rates also were observed in neonates with lower BW (*P* = 0.038), GA < 28 weeks (*P* = 0.056), however, the differences of later one were not statistically significant. We did not show it may be due to the low sample size. Further studies are needed to explore their relationship.

Some studies suggested that *L. monocytogenes* was more commonly associated with sporadic episodes and outbreaks rather than being affected by factors such as climate and season [20, 27]. However, seasonal trends of pregnancy-associated listeriosis cases have been observed in Beijing and Taiwan [18, 28], with peaks reported in summer. Similarly, in our study, more than half of pregnancy-associated listeriosis cases were occurred in summer (50.0%, 7/14). As *L. monocytogenes* was a psychrophilic organism, the ready-to-eat foods and foods stored at refrigeration temperatures are the main sources of *L. monocytogenes *[29]. And people have more opportunities to eat food that is inadequately cooked and improperly stored in the refrigerator during the warmer seasons. Therefore, they were more likely to be *L. monocytogenes* infected in summer.

Unfortunately, the incidence of pregnancy-associated listeriosis in our hospital (16.69/100,000) was higher than that in the United States, the United Kingdom, and New Zealand (3.4, 3.4 and 12.3 per 100,000 births, respectively) [19–21], where listeriosis was a notifiable disease. The information on listeriosis especially pregnancy-associated cases is limited, and this might due to the lack of a good listeriosis surveillance system in China [30]. Besides, the incidence of pregnancy-associated listeriosis in our hospital was also higher than that in one hospital in Beijing (13.7/100,000), this might be because our hospital is the only tertiary maternity and pediatric hospital in this area that admits many critically ill pregnant women and neonates.

There are several limitations to our study. First, because the miscarriage products at the first trimester GA were usually not available for microbiological investigations, and the blood cultures were not routine examinations for pregnant women without the severe performance of infections in our hospital, the occurrence of maternal listeriosis was likely to be underestimated. Second, it was a hospital-based study, the sample size was relatively small, and some information was missing (information of two mothers of outborn listeriosis neonates), so our results could not be generalized to the whole population. Third, it was a pity that we did not preserve the strains of *L monocytogenes*, molecular epidemiology data were unavailable. Therefore, a study with large sample size and relevant experiments is strongly encouraged.

## Conclusions

Although rare, pregnancy-associated listeriosis would bring disastrous effects to the neonatal cases, especially to the outborn, low birth weight, and low gestational age of neonates. Timely detection and treatment should be taken seriously for the key neonates. Meanwhile, we found that there is a neglected burden of pregnancy-associated listeriosis in our hospital, therefore, how to early detect *L. monocytogenes* infected cases, especially in the prenatal stage, remains a serious challenge.

## Data Availability

The datasets used and/or analyzed during the current study are available from the corresponding author on reasonable request.
